# The Level of Empathy Among Medical Students at the University of Tabuk, Saudi Arabia

**DOI:** 10.7759/cureus.51710

**Published:** 2024-01-05

**Authors:** Omnia S El Seifi, Amal A Alenazi, Asmaa M Alfuhaymani, Alshaymaa A Alanazi, Omayrah A Alanazi, Lama A Alanazi, Nouf M Albalawi, Fatima S Alharbi, Dhuha A ALQasir

**Affiliations:** 1 Family and Community Medicine Department, Faculty of Medicine, University of Tabuk, Tabuk, SAU; 2 Community Medicine Department, Faculty of Medicine, Zagazig University, Zagazig, EGY; 3 Internal Medicine Department, Faculty of Medicine, University of Tabuk, Tabuk, SAU; 4 Pediatrics Department, Faculty of Medicine, University of Tabuk, Tabuk, SAU

**Keywords:** university of tabuk, jefferson scale of empathy, medical students, empathy, patient-physician relationship

## Abstract

Background and objective

Empathy plays an important role in patient-provider relationships. It is a key aspect of therapy, ensuring accurate diagnosis, and improving compliance and outcomes, all of which contribute to clinician satisfaction. This study aimed to assess the level of empathy among medical students at the University of Tabuk.

Methods

A cross-sectional study was conducted among medical students at Tabuk University. Data were collected using a self-administered online questionnaire based on the Jefferson Scale of Physician Empathy-Student Version (JSPE-S).

Results

A total of 230 medical students participated in this study. The students’ total empathy scores ranged between 55 and 131, with a mean of 99.05 ± 13.75. The highest item score was obtained for the question “Patients feel better when their physicians understand their feelings“ (6.34 ± 0.99). Female students had a significantly (p=0.002) higher mean score (100.67 ± 13.06) than males (94.36 ± 14.70). Students from the clinical phase had a significantly higher mean total score compared to those from preclinical phases (100.26 ± 14.34 vs. 96.78 ± 12.33, p=0.043). Students choosing people-oriented specialties had significantly higher mean total scores than those selecting procedure-oriented specialties (100.59 ± 13.72 vs. 95.67 ± 14.46, p=0.033).

Conclusion

The degree of students’ empathy with the patients at the Faculty of Medicine, University of Tabuk was found to be highest among females, students in the clinical phase, and students intending to select people-oriented specialties. These findings have implications for medical education programs, highlighting the importance of fostering empathy skills and addressing potential gender differences in empathy development.

## Introduction

Proper communication between patients and physicians is an important aspect of medical practice. Empathy represents one of the most significant cornerstones of the patient-provider relationship [1,2]. Empathy in medical practice is defined as the physician's ability to understand the patient's attitude, point of view, feelings, and fears; communicate this understanding effectively; and verify its accuracy. Based on the patient's understanding, the physician provides the necessary care [1]. In addition, it helps physicians learn more about their patients, which is a key therapeutic aspect of therapy, as well as make accurate diagnoses and ensure good treatment compliance and outcomes, all of which contribute to physician satisfaction [3].

Several studies have confirmed the association between the physician's empathy and positive clinical outcomes. One study involving trauma surgery patients found that patients who expressed the highest rating for physician's empathy had a 20 times higher probability of experiencing better medical treatment outcomes compared to patients with physicians who had lower empathy ratings. This emphasizes the importance of empathy and a good patient-physician relationship, even in the surgical field where the focus is mainly on medical treatment [4,5].

Empathy among medical students is also an important aspect, which can be examined and sustained with the aid of educational initiatives involving lectures, showing/discussing videos of patient encounters, and focusing on empathy and communication skills training [6,7]. Student gender and academic level are among the most important factors influencing the level of empathy among undergraduate medical students [2].

Empathy has three basic components: Compassionate Care, which is the ability to feel compassion toward the patient, and depends on physiological, behavioral, cultural, and religious factors; Perspective Taking, which involves the ability to take on the patient's perspective, and is related to the physicians' ability to differentiate themselves from the patient's feelings and not to be affected by emotional contagion; Walking in Patient's Shoes, which is the ability to understand others, actively observe them, and go through their thinking [8,9]. To the best of our knowledge, there are currently no studies in the literature investigating the level of empathy among medical students at the University of Tabuk. In light of this, the current study aimed to assess the level of empathy and explore its associated factors among medical students at the University of Tabuk.

## Materials and methods

Study design and setting

This was a cross-sectional study conducted at the Faculty of Medicine, University of Tabuk, Saudi Arabia, from March to May 2023.

Inclusion and exclusion criteria

The study recruited undergraduate medical students of both genders in all academic years from the Faculty of Medicine, University of Tabuk, Saudi Arabia. Non-medical students, postgraduate students, and those with incomplete data were excluded.

Sample size calculation and sampling technique

The sample size was calculated using the Epi Info™ 7 program by assuming a percentage of empathy of 75.7% [10], a total number of 800 students, a confidence interval of 95%, and a design effect of 1.0. This resulted in a sample size of 210. By factoring in a 10% non-response rate, the final sample size was determined to be 231. A convenience sampling technique was used to recruit the study participants from the medical student body from all years.

Data collection tool

Data were collected using the standard, validated Jefferson Scale of Physician Empathy-Student Version (JSPE-S) [11], which includes 20 items measuring empathy and answered on a 7-point Likert scale (1: strongly disagree; 2: disagree; 3: somewhat disagree; 4: neither agree nor disagree; 5: somewhat agree; 6: agree; 7: strongly agree). Ten items are negatively worded and hence they are reverse-coded. Empathy has three components: Compassionate Care, represented by 11 questions, giving a range of scores from 11 to 77; Perspective Taking, assessed by seven questions, with a score range from 7 to 49; and Walking in Patient's Shoes, assessed using two questions, with a range from 2 to 14. This gives the JSPE-S a total score ranging from 20 to 140, with higher scores indicating higher levels of empathy [10,12]. Student gender, academic year, and desired future specialty were also included in the questionnaire. The questionnaire was distributed online via Google Forms. The participants' responses to the questions were complete except for one student who was excluded during data analysis.

For data analysis and interpretation, the collected data on students' future specialties were classified into people-oriented (e.g., internal medicine, obstetrics & gynecology, physical medicine, ophthalmology, psychiatry, family medicine) and procedure-oriented (e.g., surgery, radiology, anesthesiology) specialties [13]. In the Faculty of Medicine, University of Tabuk, the medical program starts with Phase I (foundation year), followed by Phase 2 (preclinical), and then Phase III (clinical) [14]. Thus, the academic year was divided into preclinical (years one, two, and three) and clinical (years four, five, and six) phases.

Ethical considerations

This study was approved by the Research Ethics Committee at the University of Tabuk, Tabuk, Saudi Arabia (approval number: UT-232-105-2023). An explanation of the purpose of the study, text ensuring the confidentiality of the participant's data, and a request for the students' consent to participate in the study were provided on the first page of the questionnaire.

Data analysis

The data were analyzed using the IBM SPSS Statistics version 22 (IBM Corp., Armonk, NY). Categorical data (such as gender, academic year, and future specialties) were summarized as counts and percentages. Numerical data (e.g., the scores for each item, or component) were summarized as the median and interquartile range (IQR: expressed as 25th-75th percentiles) for the Likert-scale data. The mean and standard deviation (SD) were used for presenting the components and the total scores. We added the mean and SD to the Likert-scale items to allow for comparison with similar previous studies that provided only these statistics. The scores were compared using the independent samples t-test (for two groups such as for gender) or one-way analysis of variance (ANOVA; for more than two groups). A p-value <0.05 was considered statistically significant.

## Results

**Figure 1:**
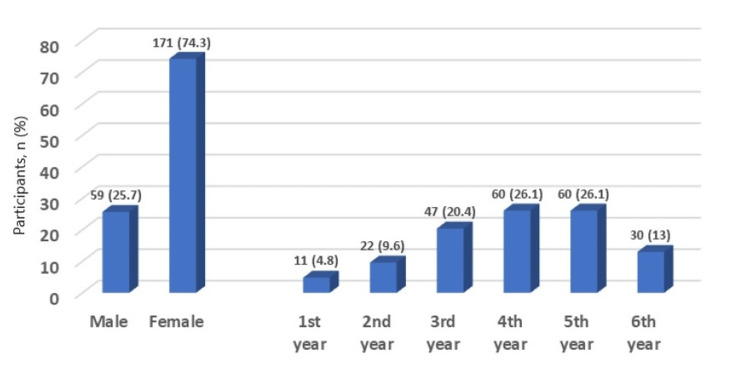
Distribution of participants by gender and academic year

A total of 230 medical students were enrolled in the study. Female students represented 74.3%, while males accounted for 25.7%. The highest participation was from fourth- and fifth-year students (26.1% each), and the lowest was from First-year students (4.8%) (Figure 1).

Regarding specialties that the enrolled medical students desired to select in the future, 91 (39.6%) students opted for people-oriented specialties, while 64 (27.8%) students selected procedure-oriented specialties. Only 75 (32.6%) students stated that they had not yet determined their future specialty (Table 1).

**Table 1:** Desired future specialty

Planned specialty	N	%
People-oriented	91	39.6
Internal medicine	19	8.3
Ophthalmology	15	6.5
Obstetrics & gynecology	14	6.1
Dermatology	11	4.8
Pediatrics	11	4.8
Family medicine	8	3.5
Psychiatry	8	3.5
Emergency medicine	2	0.9
Community medicine	2	0.9
Physical medicine	1	0.4
Procedure-oriented	64	27.8
Surgery	22	9.6
Neurosurgery	14	6.1
Radiology	8	3.5
Orthopedics	6	2.6
Anesthesia	5	2.2
ENT	4	1.7
Cardiothoracic surgery	2	0.9
Forensic medicine	2	0.9
Oncology	1	0.4
Not yet determined	75	32.6
Unknown	75	32.6

Among the responses to the JSPE-S, the items with the lowest scores were as follows: "It is difficult for a physician to view things from the patient's perspective" (3.73 ± 1.47); "Because people are different, it is difficult to see things from the patient's perspective" (3.02 ± 1.44); and "Physicians should not be influenced by strong personal bonds between their patients and their family members" (2.74 ± 1.45). The item with the highest score was "Patients feel better when their physicians understand their feelings" (6.34 ± 0.99) (Table 2).

**Table 2:** Total score of Jefferson Scale of Physician Empathy–Student Version (JSPE-S) for each question IQR: interquartile range; SD: standard deviation

No.	Questions	Mean ± SD	Median (IQR)
Q1	Physicians' understanding of their patients' feelings and the feelings of their patients' families does not influence medical or surgical treatment	4.18 ± 2.11	5 (2–6)
Q2	Patients feel better when their physicians understand their feelings	6.34 ± 0.99	7 (6–7)
Q3	It is difficult for a physician to view things from patients' perspectives	3.73 ± 1.47	4 (3–5)
Q4	Understanding body language is as important as verbal communication in physician-patient relationships	5.87 ± 1.18	6 (5–7)
Q5	A physician's sense of humor contributes to a better clinical outcome	5.46 ± 1.32	6 (5–7)
Q6	Because people are different, it is difficult to see things from patients' perspectives	3.02 ± 1.44	3 (2–4)
Q7	Attention to patients' emotions is not important in history-taking	4.90 ± 1.92	6 (4–6)
Q8	Attentiveness to patients' personal experiences does not influence treatment outcomes	4.86 ± 1.80	5 (4–6)
Q9	Physicians should try to stand in their patients' shoes when providing care to them	5.37 ± 1.42	6 (4–7)
Q10	Patients value a physician's understanding of their feelings which is therapeutic in its own right	5.78 ± 1.18	6 (5–7)
Q17	Patients' illnesses can be cured only by medical or surgical treatment; therefore, physicians' emotional ties with their patients do not have a significant influence on medical or surgical treatment	4.85 ± 1.87	5 (4–6)
Q12	Asking patients about what is happening in their personal lives is not helpful in understanding their physical complaints	4.67 ± 1.86	5 (3–6)
Q13	Physicians should try to understand what is going on in their patients' minds by paying attention to their non-verbal cues and body language	5.58 ± 1.32	6 (5–7)
Q14	I believe that emotion has no place in the treatment of medical illness	5.21 ± 1.77	6 (4–7)
Q15	Empathy is a therapeutic skill without which the physician's success is limited	4.87 ± 1.61	5 (4–6)
Q16	Physicians' understanding of the emotional status of their patients, as well as that of their families, is one important component of the physician-patient relationship	5.84 ± 1.22	6 (5–7)
Q17	Physicians should try to think like their patients in order to render better care	5.37 ± 1.33	6 (4–6)
Q18	Physicians should not allow themselves to be influenced by strong personal bonds between their patients and their family members	2.74 ± 1.45	3 (2–4)
Q19	I do not enjoy reading non-medical literature or the arts	4.67 ± 1.89	5 (3–6)
Q20	I believe that empathy is an important therapeutic factor in medical treatment	5.74 ± 1.30	6 (5–7)

The mean scores of the three components of the JSPE-S (Compassionate Care, Perspective Taking, and Walking in Patient's Shoes) were as follows: 56.14 ± 10.84, 36.16 ± 4.87, and 6.75 ± 2.53, respectively. The mean total empathy score was 99.05 ± 13.75 (Table 3).

**Table 3:** Scores for empathy and its components among participants The students’ total empathy scores ranged between 55 and 131 SD: standard deviation

Empathy components	Questions	Minimum	Maximum	Mean ± SD
Compassionate Care	1, 2, 7, 8, 11, 12, 14, 15, 16, 19, 20	24	76	56.14 ± 10.84
Perspective Taking	4, 5, 9, 10, 13, 17, 18	23	47	36.16 ± 4.87
Walking in Patient’s Shoes	3, 6	2	14	6.75 ± 2.53
Total empathy score	All questions	55	131	99.05 ± 13.75

Female students had higher mean total empathy scores than their male counterparts, and this difference was statistically significant (100.67 ± 13.06 vs. 94.36 ± 14.70, p=0.002). There were no significant differences in the mean total empathy scores of students from different academic years (p=0.099). However, when the academic years were classified into two categories, phase III students had significantly higher mean total empathy scores than preclinical students (100.26 ± 14.34 vs. 96.78 ± 12.33, p=0.043). Also, the group of students choosing people-oriented specialties had significantly higher mean total scores (100.59 ± 13.72 vs. 95.67 ± 14.46, p=0.033). (Table 4).

**Table 4:** Comparison of empathy scores among participants by gender and academic year *Statistically significant p-value F: one-way analysis of variance (ANOVA) test; SD: standard deviation; t: Independent samples t-test; Z: Mann-Whitney U test

	N	Mean ± SD	Test value	P-value
Gender				
Female	171	100.67 ± 13.06	t=3.096	0.002*
Male	59	94.36 ± 14.70		
Academic year				
First year	11	102.55 ± 7.59	Z=9.265	0.099
Second year	22	94.77 ± 13.19		
Third year	47	96.36 ± 12.62		
Fourth year	60	100.47 ± 15.39		
Fifth year	60	101.63 ± 13.21		
Sixth year	30	97.10 ± 14.32		
Academic phase				
Preclinical (Foundation year and Phase II)	80	96.78 ± 12.33	Z=2.028	0.043*
Clinical (Phase III)	150	100.26 ± 14.34		
Specialty				
People-oriented	91	100.59 ± 13.72	t=2.150	0.033*
Procedure-oriented	64	95.67 ± 14.46		

## Discussion

This study aimed to assess empathy among medical students at the Faculty of Medicine, University of Tabuk, and it involved 231 students; one of them was excluded during data analysis due to incomplete responses to the questionnaire.

In terms of gender distribution, female students had a higher participation rate (74.3%) compared to male students (25.75%). This is consistent with the findings of previous studies [2,15-19], which also reported a higher participation rate among female students. The current study also found that fourth- and fifth-year students had a higher response rate, while first-year students had the lowest response rate. This may be because senior students are more involved in clinical practice than junior students, which makes senior students more interested and motivated to participate in the study. However, Chen et al. [10] reported a higher response rate among first- and second-year students, while third-year students had the lowest response rate, which may be due to their academic responsibilities at the time of the survey.

The use of the Jefferson Scale of Empathy provided further insights into specific beliefs held by medical students. The item “Patients feel better when their physicians understand their feelings” received the highest mean empathy score (6.34 ± 0.99). This finding highlights the importance of recognizing the role of emotions in patient care and the potential impact on empathy levels among medical students. It emphasizes the need for educational interventions that promote empathy and understanding of patients' emotional experiences.

The mean total empathy score in our cohort was 99.05 ± 13.75, which is close to the reported total empathy score in comparable studies. Studies from India reported mean total empathy scores of 96.01 ± 14.56 [20] and 99.87 ± 14.71 [21] among undergraduate students from medical colleges in Delhi and Bihar, respectively, whereas two studies from Sukkur and Karachi, Pakistan, reported mean empathy scores of 98.11 ± 12.31 [12] and 101.9 ± 16.3 [22], respectively. Some studies have reported higher mean total empathy scores among medical students, such as those from Australia (109.07 ± 14.937) [23], South Korea (105.48 ± 14.67) [24], and Brazil (119.7 ± 9.9) [25]. These differences may be due to the different educational settings in each country.

A difference was observed between male and female students, with female students having significantly higher empathy scores (100.67 ± 13.06) compared to male students (94.36 ± 14.70). This suggests that gender may play a role in empathy levels among medical students. This finding aligns with previous studies [2,3,12,20]. The difference between the two genders can be attributed to psychological and emotional differences between them, as well as integrating these emotions into caring for patients [12].

Regarding the comparison of mean empathy scores across the six academic years, the present study found no significant differences. These findings are consistent with those of previous studies [21-23]. However, our results contrast with those of Kataoka et al. [26], who reported significantly higher mean total scores for students in years two, three, five, and six compared to those in year one. Meanwhile, Park et al. [24] found that the significant difference existed only in second-year students, who had lower scores than first-year students. Chatterjee et al. [20] and Mirani et al. [12] observed significant differences between academic years, but the empathy scores decreased in higher years compared to the first year, which had the highest score.

When comparing the total empathy scores between the educational phases (preclinical vs. clinical phases), we found that the clinical phase students had a significantly higher total empathy score compared to the preclinical phase students. These results may be attributed to the fact that senior students, who have more contact with patients, may be affected by their encounters with patients and begin to appreciate the importance of fostering their relationship with patients, realizing that empathy is an important cornerstone in this relationship. It should be noted that we combined the foundation year with phase I and analyzed it as the preclinical phase due to the low number of students participating from the first year. Kataoka et al. [26] found an increase in clinical empathy with the increasing number of years of education, whereas Shashikumar et al. [27] found a decreasing trend over the years. The variation in empathy levels between different academic years suggests that the sense of empathy may develop and evolve throughout medical education [20].

The present study also found that the mean total empathy score was significantly higher for students who intended to specialize in people-oriented fields compared to those who desired to specialize in procedure-oriented specialties. This finding is consistent with previous studies that have examined this relationship [10,12,21,25,28,29]. However, in the study by Chatterjee et al. [20], the difference was marginally non-significant. Higher empathy scores among students who prefer people-oriented specialties seem intuitive and expected. These students are assumed to understand patients' feelings and appreciate the value of this trait in the doctor-patient relationship, and they are naturally expected to be attracted to specialties that put them in close contact with patients.

Overall, we believe this study contributes to the understanding of empathy levels among medical students at the University of Tabuk. The findings suggest that gender, phase of medical study, and desired future specialty may influence empathy levels, and specific beliefs about the role of emotions in medical treatment may influence empathy scores. These findings have implications for medical education programs, highlighting the importance of fostering empathy skills and addressing potential gender differences in empathy development.

This study has a few limitations. Apart from its single-center setting, the convenience sampling technique used to recruit participants in this study may affect the generalizability of the findings. Also, the self-reporting nature of the data may lead to overestimation or underestimation of empathy, which may introduce an element of bias in the study results.

## Conclusions

This study found that the level of students’ empathy with the patients at the Faculty of Medicine, University of Tabuk was highest among females, students in the clinical phase, and students intending to choose people-oriented specialties in the future. To ensure that future physicians have the skills necessary to provide effective patient care, further research is needed to explore the factors that underlie these findings and to develop targeted interventions throughout medical education.
